# Rise of the Machines: WATCHMAN Device Gone Rogue

**DOI:** 10.1016/j.acepjo.2026.100332

**Published:** 2026-02-07

**Authors:** Jaclyn Angielczyk, Jessica Sidle, Zachary Webb, Edward Perez

**Affiliations:** Department of Emergency Medicine, Huntington Hospital, Huntington, New York, USA

A 68-year-old man with a past medical history of atrial fibrillation with ablation and WATCHMAN procedure performed 5 days prior presented with bilateral lower extremity numbness, right leg weakness, and low back pain. His examination was significant for midline lumbar spine tenderness in the L1 to L2 distribution, diminished strength in the right lower extremity, and decreased sensation in his bilateral lower extremities. Computed tomography of the lumbar spine was performed with contrast, which demonstrated embolization of the WATCHMAN device to the abdominal aorta, between the celiac axis and the superior mesenteric artery (Fig.)

**Figure:**
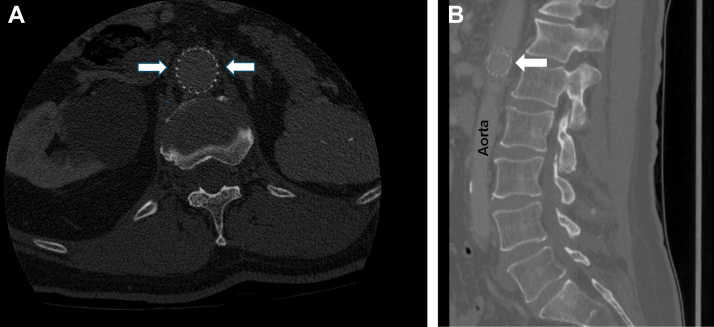
Coronal (A) and sagittal (B) computed tomography images demonstrating the presence of a WATCHMAN device (arrows) within the abdominal aorta (labeled).

In patients with atrial fibrillation, the WATCHMAN device is inserted via catheterization into the left atrial appendage to decrease thromboembolism formation. This has shown utility in patients who are poor candidates for lifelong anticoagulation use due to increased risk of bleeding.^1^

Although left atrial appendage occlusion devices have proven effective in stroke prevention, device embolization remains a rare but serious complication. Reported embolization rates vary between 0.25% and 0.7% depending on the device generation and trial population, with 0.2% occurring within 7 days of implantation.^2^, ^3^, ^4^

Embolization can occur at multiple sites, such as the left atrium, left ventricle, and aorta.^2^^,^^4^ Migration to the left atrium or ventricle presents as heart failure and can be life threatening. If the device embolizes into the thoracic or abdominal aorta, it may be clinically silent but can present similarly to spinal pathologies or aortic dissection with limb weakness, back pain, and sensation discrepancies.^1^ Retrieval success and outcomes does vary by location, with percutaneous retrieval often successful; whereas, surgical intervention is more common in left ventricular cases.^4^

## Funding and Support

By *JACEP Open* policy, all authors are required to disclose any and all commercial, financial, and other relationships in any way related to the subject of this article as per ICMJE conflict of interest guidelines (see www.icmje.org). The authors have stated that no such relationships exist.

## Conflicts of Interest

All authors have affirmed they have no conflicts of interest to declare.
